# A control mechanism for intra-mural peri-arterial drainage via astrocytes: How neuronal activity could improve waste clearance from the brain

**DOI:** 10.1371/journal.pone.0205276

**Published:** 2018-10-04

**Authors:** Alexandra K. Diem, Roxana O. Carare, Roy O. Weller, Neil W. Bressloff

**Affiliations:** 1 Department of Computational Physiology, Simula Research Laboratory, 1364 Fornebu, Norway; 2 Computational Engineering and Design, Faculty of Engineering and the Environment, University of Southampton, Southampton Boldrewood Innovation Campus, Southampton, SO16 7QF, United Kingdom; 3 Clinical Neurosciences, Faculty of Medicine, University of Southampton, Southampton General Hospital, Southampton, SO16 6YD, United Kingdom; 4 Neuropathology, Southampton General Hospital, Southampton, SO16 6YD, United Kingdom; Hungarian Academy of Sciences, HUNGARY

## Abstract

The mechanisms behind the clearance of soluble waste from deep within the parenchyma of the brain remain unclear. Experimental evidence reveals that one pathway for clearance of waste, termed intra-mural peri-arterial drainage (IPAD), is the rapid drainage of interstitial fluid along basement membranes (BM) of the smooth muscle cells of cerebral arteries; failure of IPAD is closely associated with the pathology of Alzheimer’s disease (AD), but its driving mechanism remains unclear. We have previously shown that arterial pulsations generated by the heart beat are not strong enough to drive IPAD. Here we present computational evidence for a mechanism for clearance of waste from the brain that is driven by functional hyperaemia, that is, the dilatation of cerebral arterioles as a consequence of increased nutrient demand from neurons. This mechanism is based on our model for the flow of fluid through the vascular BM. It accounts for clearance rates observed in mouse experiments, and aligns with pathological observations and recommendations to lower the individual risk of AD, such as mental and physical activity. Thus, our neurovascular hypothesis should act as the new working hypothesis for the driving force behind IPAD.

## Introduction

The brain lacks traditional lymphatics and until now the driving mechanisms behind the clearance of soluble waste from the brain have remained largely unclear. However, pathological evidence suggests the presence of a pathway for the clearance of soluble waste along the basement membranes (BM) of smooth muscle cells (SMC) within artery walls, termed intramural peri-arterial drainage (IPAD). Failure of IPAD occurs as cerebral arteries become stiffer with age [1–3] and is closely associated with the pathological accumulation of amyloid-β (Aβ) [4]. This has lead to the hypothesis that arterial pulsations generated by the heart drive the clearance of soluble waste from the brain [5–8]. In our previous study [9] we used a combination of computational modelling and experiments to investigate this hypothesis. Whilst we did not measure mean arterial blood pressure before and after treatment, our combination of *in silico* and *in vivo* experiments demonstrate that arterial pulsations are not strong enough to drive IPAD and thus the exact mechanisms behind clearance of waste from the brain by this pathway remain unclear [9]. Computational models, when developed in close collaboration with clinical experts, can be an excellent tool for pre-clinical research. This view is backed by a large number of experts in particular in small vessel disease and dementia [10]. We extend this idea here to develop a new hypothesis of how soluble waste may be driven from the brain under the IPAD hypothesis.

**Fig 1:**
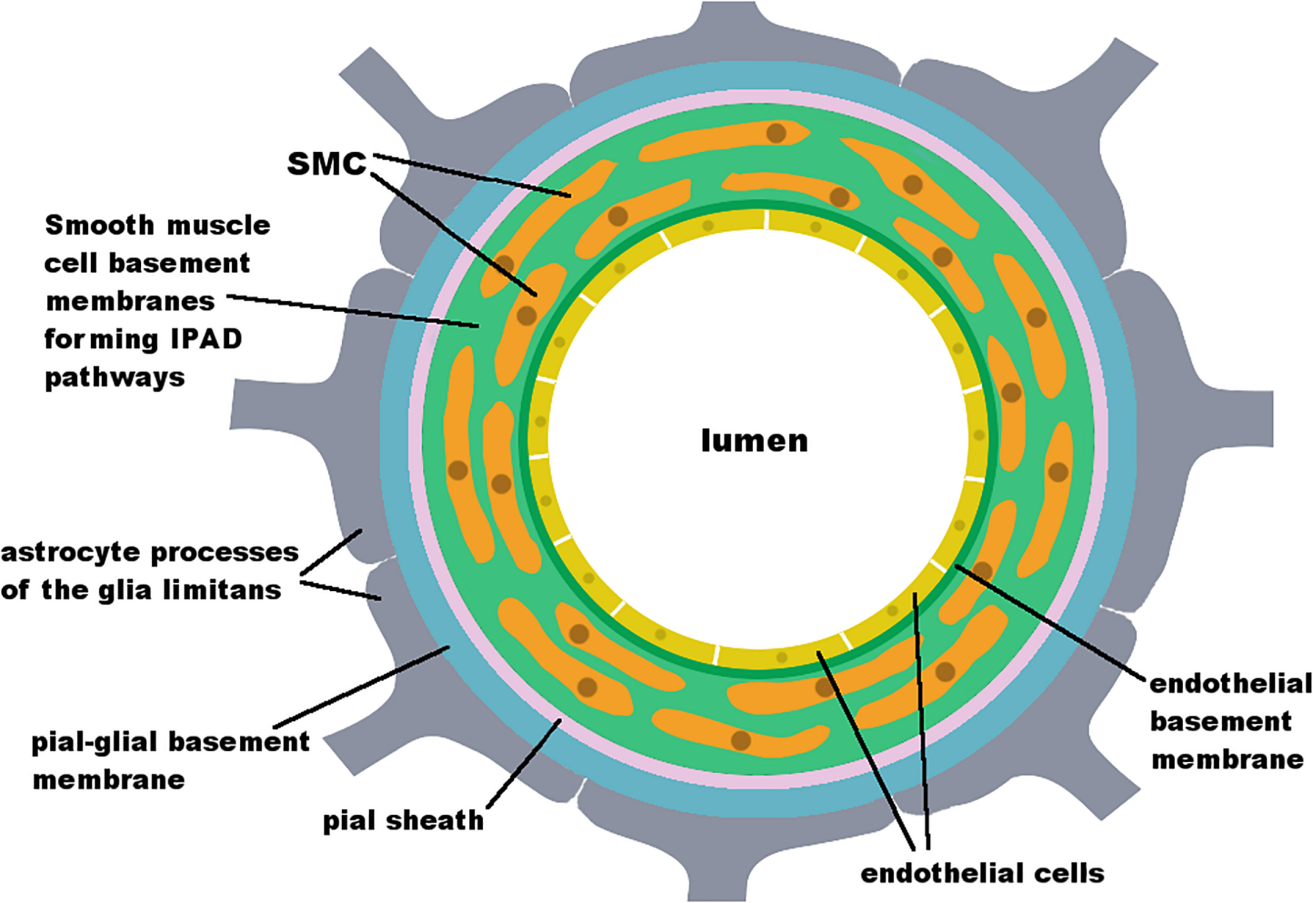
Schematic diagram of a cross-section of a cerebral artery demonstrating the geometry and location of the IPAD pathway. The artery lumen is surrounded by an endothelium (yellow), which forms a BM (dark green). Several layers of SMC, which express their own type of BM (light green) surround the artery. A pial or leptomeningeal sheath (pink), which is derived from the pia mater, surrounds the outside of the artery. Arteries are covered tightly by astrocyte end-feet (grey), forming the pial-glial BM (glia limitans) [11, 14, 15].

The pathway of IPAD was first described by Carare et al. [1] and is one of two major hypotheses on the mechanism behind the clearance of soluble waste from the brain. Its geometry and location is shown in Fig 1 and further detailed in [11]. IPAD occurs through the BM of the SMC, which consists of extracellular matrix, a tight network of proteins. A recent molecular characterisation of the vascular BM in the murine brain has been published by Hannocks et al. [12]. Other hypotheses on the exact pathway of soluble waste clearance exist and we refer the reader to the review by Hladky and Barrand [13] for an overview over the debate.

Here, we use computational simulations to demonstrate how functional hyperaemia, with dilatation of cerebral arteries due to an increase in neuronal activity, may provide the driving force for clearance of soluble waste material from the brain. Chemical communication within the neurovascular unit (NVU), which comprises neurons, astrocytes and arteries [16], can lead to a dilatation of 20% of a small artery of around 40 μm diameter [17, 18]. Data by [19], who have measured cerebral blood flow regulation, agrees with this estimate. The model that is proposed here is an extension of our previous model. In our extension, we have combined our previous model for IPAD with a model of functional hyperaemia in order to generate a better hypothesis for IPAD and hence suggest potential mechanisms that may lead to the development of Alzheimer’s disease (AD). We successfully demonstrate the clearance of waste metabolites from the brain at a rate comparable to experimental observations by [1] by combining our model of flow through the BM by [9] with a finite element (FE) model of the artery wall of an arteriole. Throughout the manuscript we use the term “artery” when describing concepts and methods that apply to both larger arteries and smaller arteries, i. e. arterioles, and we used the term “arteriole” when referring to our specific simulation. In addition we provide a potential explanation how this mechanism is impaired by stiffening of the artery wall, as commonly occurs with age and leads to risk factors for AD such as arteriosclerosis and atherosclerosis [20]. Our suggested mechanism is in line with key pathological features of AD and with recommendations to reduce the individual risk of developing AD via physical and mental activity as issued by the Alzheimer’s Society [21]. Additionally our hypothesis is supported by the findings of [22] whose experimental results suggesting a correlation between mild-to-moderate AD and damaged neurovascular coupling.

## Model of functional hyperaemia

**Fig 2:**
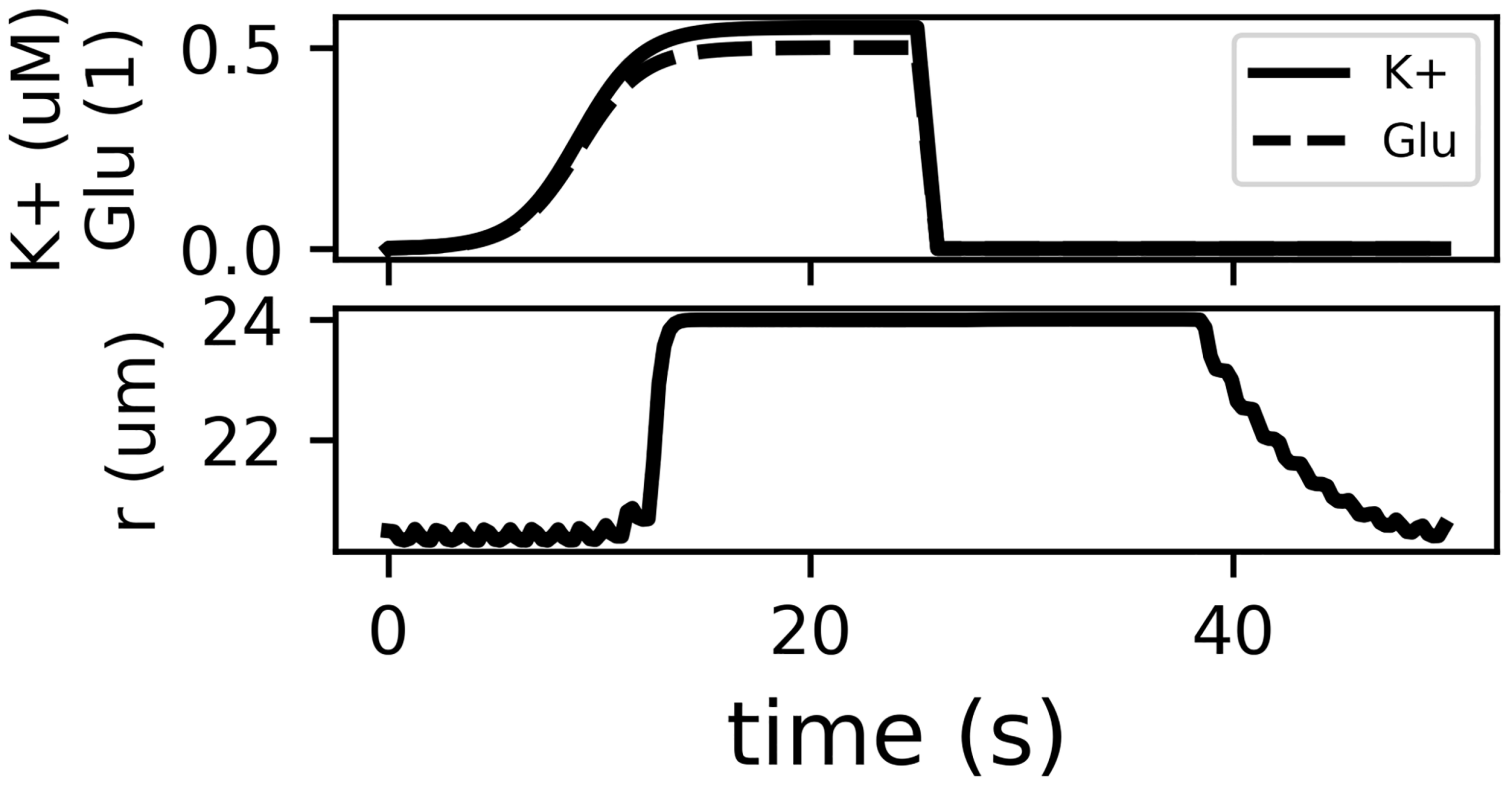
Effect of the release of K^+^ and Glu into the synaptic space of a neuron and an astrocyte process. Glu here refers to the ratio of bound/unbound Glu receptors (dimensionless). The arterial radius is modelled using a system of ODE [18] with corrections to the equations listed in [24]. The model shows dilatation of the artery of 20%. This figure only shows the input (a) and result (b) of the functional hyperaemia model. We refer the reader to the original and reimplementation publications for the full details of the chemical cascade implemented in the model and the source code.

The brain is the fastest metabolising organ in the body and has unique energy demands. Whilst only taking up 2% of our body mass it consumes 20% of our energy [23]. Thus, adequate supply of nutrients to neurons is vital for maintaining brain function. Functional hyperaemia is the mechanism by which the NVU increases blood flow to an active region of the brain. The synapses of active neurons release glutamate (Glu) and potassium (K^+^), which, following a cascade of ionic signalling, leads to the release of internal Ca^2+^ stores from the astrocyte via its endfeet. The SMC membrane is depolarised and the artery dilates [16]. This chemical signalling cascade has been described and modelled using ordinary differential equations (ODE) by [18]. We have reimplemented this model and refer the reader to the original publication [18] and our open access publication of our reimplementation [24] for the full details of the chemical processes and model description. Fig 2 shows the model input (a, Glu and K+ release at the neuron-astrocyte synaptic connection) and the resulting arterial dilatation (b). The displacement *U*(*t*) of the artery is defined as

$$\begin{array}{c} U\left(t\right)=r\left(t\right)-r_{0}, \end{array}$$ (1)

where *r*_0_ is the arterial radius at rest.

We modelled an artery wall based on an arteriole with radius *r*_0_ = 20 μm as a two-dimensional rectangle of height *h* = 4 μm and length *l* = 200 μm. To the best of our knowledge it is impossible to estimate exactly how long an arteriole of a certain radius should be. The length here was chosen such that it is sufficient for the effects of the dilatation to have vanished at the boundaries. Similarly, we could not find recent references for the width of an astrocyte end-foot. A single study from 1985 estimates its width as at least 4 μm [25]. We have chosen a width of 10 μm, noting that pilot results indicated that a shorter width would result in faster flow rates. That means that our model is more likely to underestimate flow rates through the BM.

We developed our model using the FE solver FEniCS [26], using a linear elastic model with the governing equations

$$\begin{array}{c} -\nabla \cdot \sigma =0\;\text{on}\;\Omega \end{array}$$ (2)

$$\begin{array}{c} \sigma =\lambda \left(\nabla \cdot u\right)I+\mu (\nabla u+{\left(\nabla u\right)}^{T}), \end{array}$$ (3)

where Ω describes the domain, *u* is the artery wall displacement, *σ* is the stress tensor, *I* is the identity matrix and λ = 16.44 MPa and *μ* = 335.57 kPa are the Lamé coefficients. The radial boundaries were fixed in space while the longitudinal boundaries were allowed to deform freely. Additionally, an astrocyte was placed centrally on the outer longitudinal boundary, applying the Dirichlet boundary condition

$$\begin{array}{c} u=U\left(t\right)\;\text{on}\;\partial \Omega_{A}, \end{array}$$ (4)

where Ω_*A*_ refers to a section of the outer longitudinal boundary, which models an astrocyte. The optimal mesh consisted of 33,348 triangular elements. The governing equations were solved for 50 s with a time step of 0.25 s, where at each time step the boundary condition (4) was updated.

The rate of IPAD *q* = (*q*_1_, *q*_2_) was calculated using our model based on Darcy’s law for porous media under thin-film flow conditions [9] in cylindrical coordinates is governed by

$$\begin{array}{c} q=q_{1}e_{z}+q_{2}e_{r}=-K\left(\frac{\partial p}{\partial z}\right)\left(\frac{\partial p}{\partial z}e_{z}+\frac{\partial p}{\partial r}e_{r}\right) \end{array}$$ (5)

$$\begin{array}{c} \frac{\partial q_{1}}{\partial z}+\frac{1}{r}\frac{\partial }{\partial r}(rq_{2})=0, \end{array}$$ (6)

where ***e***_***z***_ and ***e***_***r***_ describe the unit vectors in the *z* and *r* direction and *K*(∂*p*/∂*z*) describes the permeability of the BM as a function of the gradient of pressure *p* in the *z*-direction, ∂*p*/∂*z*. Permeability is modelled as a step function

$$\begin{array}{c} K\left(\frac{\partial p}{\partial z}\right)=\frac{k}{\mu }\cdot \{\begin{array}{cc} K_{0} & \text{if}\;\partial p/\partial z<0,\\ K_{1} & \text{otherwise,} \end{array} \end{array}$$ (7)

where *k* = 1 × 10^−2^ μm^2^ − 1 × 10^−6^ μm^2^ represents the intrinsic permeability of the extracellular matrix [27], *μ* = 1.5 × 10^−3^ Pa s represents the viscosity of ISF [28] and the ratio 0 < *K*_0_/*K*_1_ ≤ 1.0 determines the strength of the valve-like mechanism as proposed in [9]. Modelling permeability as having a valve-like mechanism by utilising a step-function in ∂*p*/∂*z* is necessary to achieve net reverse flow of ISF compared to the blood flow. A smaller ratio of *K*_0_/*K*_1_ corresponds to a stronger valve-like mechanism, where positive pressure gradients encounter a smaller permeability than pressure gradients in the negative direction. Applying thin-film flow conditions and kinematic boundary conditions the model equation

$$\begin{array}{c} \frac{\partial }{\partial t}(\gamma \cdot R_{i}\left(z,t\right)\cdot h\left(z,t\right))=\frac{\partial }{\partial z}(R_{i}\left(z,t\right)\cdot h\left(z,t\right)\cdot K\left(\frac{\partial p}{\partial z}\right)\frac{\partial p}{\partial z}) \end{array}$$ (8)

is derived (see [9] for details). The IPAD model requires the pressure gradient along the artery ∂*p*/∂*z*, which can be recovered directly from the stresses inside the artery wall, such that

$$\begin{array}{c} p=-\sigma_{rr}, \end{array}$$ (9)

where *σ*_*rr*_ describes the principal component of *σ* in the *r*-direction [29].

## Results

**Fig 3:**
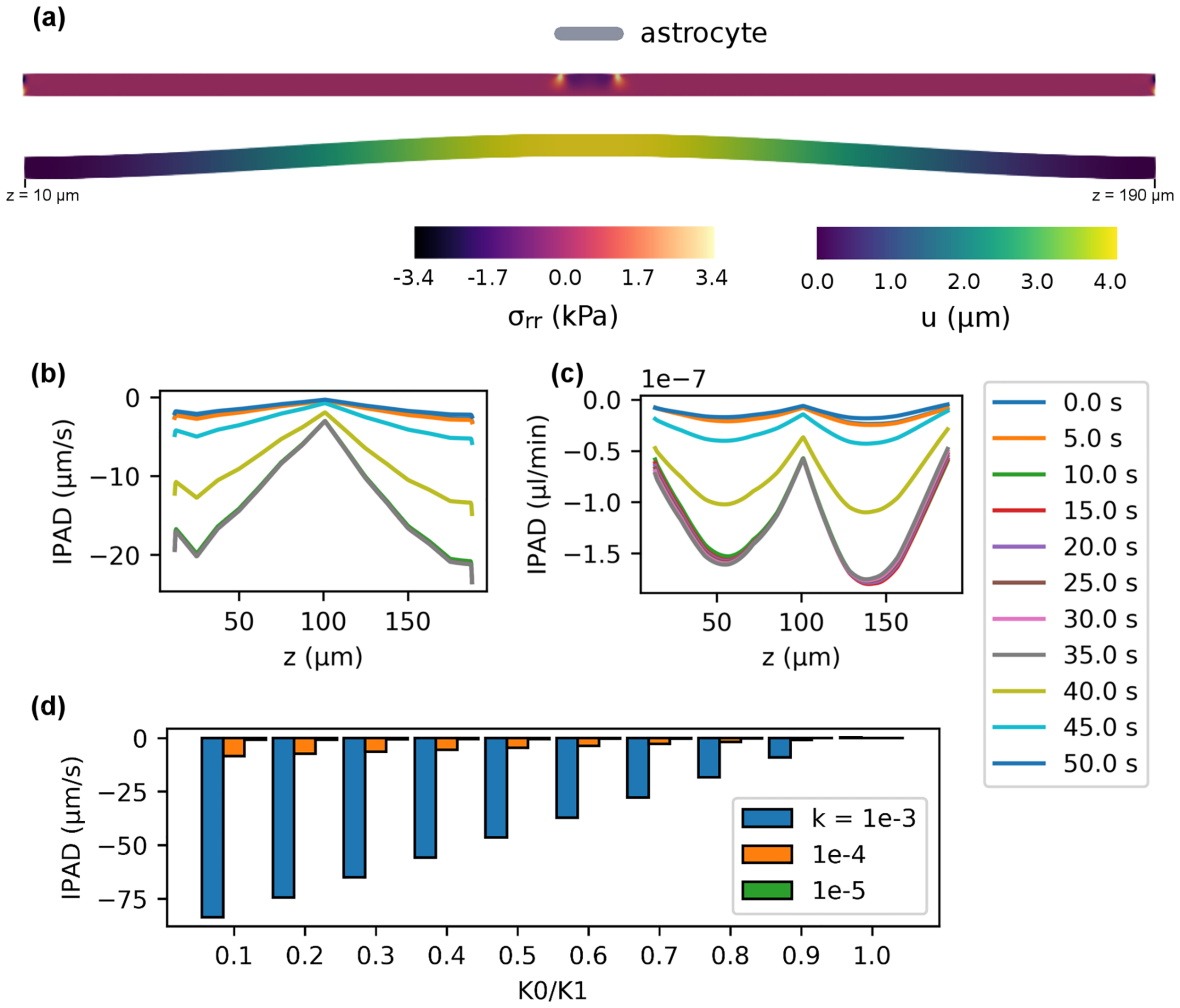
IPAD inside a cerebral arteriole. (a) Displacement and stress of the arteriole wall due to *U*(*t*) at *t* = 20 s of a single astrocyte end-foot. Because displacement is fixed to *u* = 0 at the ends, stresses at the ends are high. Thus, all following results are presented for 10 μm ≤ *z* ≤ 190 μm. (b) IPAD velocity at various time points over the length of the arteriole wall using *K*_0_/*K*_1_ = 0.1. The average velocity over time and space is −8.37 μm s^−1^. (c) IPAD flow rate at various time points over the length of the arteriole wall using *K*_0_/*K*_1_ = 0.1. The average flow rate over time and space is −7.24 × 10^−8^ μl/min for a single arteriole. Extrapolated over 6.5 billion arterioles estimated for the human brain it would take 9.92 h to process the total amount of ISF in the brain (280 ml). (d) IPAD velocity for *k* = 1 × 10^−3^ (blue), 1 × 10^−4^ (orange), 1 × 10^−5^ μm^2^ (green) over the strength of the valve mechanisms *K*_0_/*K*_1_. Values are always negative, except at *K*_0_/*K*_1_ = 1.0. The effect of the valve mechanism is decreased with decreasing *k*.

The results are shown in Fig 3. Displacement and stress inside the arteriole wall are shown (Fig 3a) for a single astrocyte of length 10 μm at *t* = 20 s, where maximal dilatation of the arteriole has been achieved (see Fig 2). The stress plot shows high stresses at both ends of the arteriole, which are due to the wall being fixed to *u* = 0. Thus, to avoid an influence of these boundary effects on the results all further results are presented for 10 μm ≤ *z* ≤ 190 μm. Experimental results from [1] suggest an expected IPAD velocity of 8 μm s^−1^ for the mouse brain, which is adopted as a benchmark value here. Using *k* = 1 × 10^−4^ μm^2^ and *K*_0_/*K*_1_ = 0.1 an average IPAD velocity of −8.37 μm s^−1^ is achieved, where the negative sign indicates flow in the reverse direction to the blood flow as is the case based on the IPAD hypothesis (Fig 3b). The average flow rate for the same parameters is −7.24 × 10^−8^ μl/min for a single arteriole (Fig 3c).

In order to decide whether the flow rates obtained by our model are capable of explaining the rapid drainage of ISF under the IPAD hypothesis we require an estimate of the total number of arterioles in the brain. We have not been able to find an appropriate estimate for arterioles in the literature, however, it is estimated that the human brain contains a total capillary length of 400 miles (∼ 644 km) [30]. Thus, we can estimate the turnover rate through the total number of capillaries instead. Assuming that the average length of a capillary is ≈ 100 μm the total number of capillaries in the brain is 6.5 billion. Thus, it would take approximately 9.92 h to process the full volume of ISF (280 ml). In comparison CSF has a turnover rate of three to four times a day [13].

Permeability *k* of extracellular matrix in the brain, and in particular the extracellular matrix that creates the BM of vascular SMC, is not known, but it ranges between 0.01 μm^2^ and 1 × 10^−6^ μm^2^ for interstitial spaces elsewhere in the body [27]. Thus it is interesting to observe IPAD velocities for different values of *k* and valve strength (Fig 3d). Using *k* = 1 × 10^−4^ μm^2^ resulted in the experimentally observed velocity of ≈ 8 μm s^−1^, and net velocity is always negative except for *K*_0_/*K*_1_ = 1.0, i. e. absence of a valve mechanism. A change of the permeability by one order of magnitude results in the same change in the IPAD velocity, thus the effect of the valve mechanism decreases with decreasing *k*.

**Fig 4:**
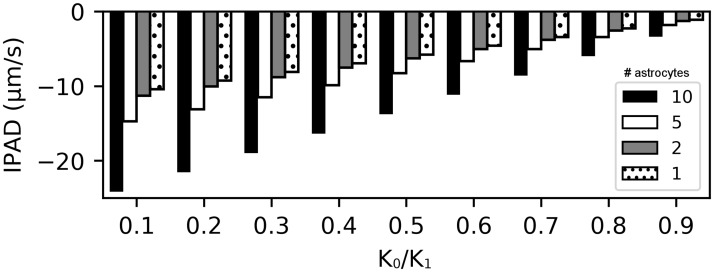
Comparison of IPAD velocities in an arteriole for varying numbers of astrocytes acting on the wall and strength of the valve-like mechanism. An astrocyte end-foot is modelled with a width of 10 μm and gap between astrocytes 1 μm. Length has a negative effect on IPAD whilst the number of astrocytes acting on the arteriole simultaneously has a positive effect. *k* = 1 × 10^−3^ μm^2^, arteriole length *l* = 309 μm, number of astrocytes: 10 (black), 5 (white), 2 (gray), 1 (dotted).

Fig 4 shows a comparison of IPAD for varying numbers of astrocytes on a longer arteriole with *l* = 309 μm. The increase in length was necessary such that the increased length of the portion of the arteriole that is influenced by astrocytes does not lead to boundary effects. Astrocyte end-feet are modelled to have a width of 10 μm with 1 μm gaps in between. Increasing the arteriole length leads to a decrease in IPAD, whilst maintaining a velocity comparable to experimentally observed values. Increasing the number of astrocytes increases IPAD. It is interesting to note that IPAD is not necessarily positive at *K*_0_/*K*_1_ = 1.0, however, if it remains negative its absolute value is more than an order of magnitude smaller compared to *K*_0_/*K*_1_ = 0.1, indicating stagnating flow. These values are not shown in Fig 4 as the values are so small that they are not visible in the plot. The dilatation of the arteriole here is not caused by a travelling wave and hence directionality of ISF flow may not be dictated by the dilatation on its own. Removing the gaps between astrocytes leads to a reduction of IPAD, which, for ten astrocytes at *K*_0_/*K*_1_ = 0.1 reduces from −24.16 μm s^−1^ to −22.06 μm s^−1^.

## Discussion

Here we have combined our previously developed model for IPAD [9] with a model of functional hyperaemia [18]. This has been done in order to develop a better hypothesis for the mechanism behind IPAD, rather than the well-cited hypothesis that arterial pulsations drive fluid and solutes along the IPAD pathways. All parameters chosen lie well within physiologically relevant ranges found in the literature. The mechanism produces velocities in accordance with experimental results from [1] and the total estimated flow rate suggests a turnover of ISF of just under three times a day, similar to the turnover rate for CSF.

Our study supports the new hypothesis that arterial dilatations due to neurovascular coupling may play a crucial role in clearance of waste from the brain. Our hypothesis is additionally supported by the recent findings of [22] whose experimental results suggest a correlation between mild-to-moderate AD and damaged neurovascular coupling. We believe that our hypothesis is more intuitive than the previously accepted hypothesis of pulsations due to the heart beat driving clearance of waste: Athletes have a comparatively low heart rate, which would put them at increased risk of developing AD under the heart beat hypothesis. However, research shows that regular physical activity acts as a preventative mechanism [31] and is generally beneficial for cognitive ability [32]. It decreases amyloid load in transgenic mouse models [33] and reduces the risk of hippocampal atrophy in individuals with a genetic risk of developing AD [34]. Our neurovascular coupling hypothesis supports and can explain these findings. Physical activity leads to increased neuronal activity, which demands better supply of nutrients and, according to our model, is accompanied by increased IPAD and thus decreased amyloid load. By analogy, the same recommendation applies more generally for any type of activity that engages cognition [35–37] and is equally well explained by our hypothesis and supported by our model coupling neuronal activity with IPAD. In addition our hypothesis supports the findings that cardiovascular diseases such as atherosclerosis and hypertension pose risk factors for AD [20], most probably via two mechanisms: (a) decreased response to astrocyte activity due to a stiffening of the arteriole wall and (b) stagnation of IPAD due to a possible dysfunction of the valve-like mechanism.

We conclude that our neurovascular hypothesis for the mechanism behind IPAD offers suitable explanations for cardiovascular findings associated with AD and should thus act as the new working hypothesis for the driving force behind IPAD.
